# The Clean pilot study: evaluation of an environmental hygiene intervention bundle in three Tanzanian hospitals

**DOI:** 10.1186/s13756-020-00866-8

**Published:** 2021-01-07

**Authors:** Giorgia Gon, Abdunoor M. Kabanywanyi, Petri Blinkhoff, Simon Cousens, Stephanie J. Dancer, Wendy J. Graham, Joseph Hokororo, Fatuma Manzi, Tanya Marchant, Dickson Mkoka, Emma Morrison, Sarah Mswata, Shefali Oza, Loveday Penn-Kekana, Yovitha Sedekia, Sandra Virgo, Susannah Woodd, Alexander M. Aiken

**Affiliations:** 1 Department of Infectious Disease Epidemiology, London School of Hygiene and Tropical Medicine, London, UK; 2 Ifakara Health Institute, Dar es Salaam, Tanzania; 3 School of Applied Sciences, Edinburgh Napier University, Edinburgh, UK; 4 Department of Microbiology, Hairmyres Hospital, Glasgow, UK; 5 Ministry of Health Community Development Gender Elderly and Children, Dar es Salaam, Tanzania; 6 Department of Disease Control, London School of Hygiene and Tropical Medicine, Dar es Salaam, Tanzania; 7 School of Nursing, Muhimbili University of Health and Allied Sciences, Dar es Salaam, Tanzania; 8 The Soapbox Collaborative, Aberdeen, UK; 9 Kent University, Canterbury, UK

**Keywords:** Environmental hygiene, Cleaning, Maternity, Training, Intervention, Pilot

## Abstract

**Background:**

Healthcare associated infections (HAI) are estimated to affect up to 15% of hospital inpatients in low-income countries (LICs). A critical but often neglected aspect of HAI prevention is basic environmental hygiene, particularly surface cleaning and linen management. TEACH CLEAN is an educational intervention aimed at improving environmental hygiene. We evaluated the effectiveness of this intervention in a pilot study in three high-volume maternity and newborn units in Dar es Salaam, Tanzania.

**Methods:**

This study design prospectively evaluated the intervention as a whole, and offered a before-and-after comparison of the impact of the main training. We measured changes in microbiological cleanliness [Aerobic Colony Counts (ACC) and presence of *Staphylococcus aureus*] using dipslides, and physical cleaning action using gel dots. These were analysed with descriptive statistics and logistic regression models. We used qualitative (focus group discussions, in-depth interviews, and semi-structured observation) and quantitative (observation checklist) tools to measure why and how the intervention worked. We describe these findings across the themes of adaptation, fidelity, dose, reach and context.

**Results:**

Microbiological cleanliness improved during the study period (ACC pre-training: 19%; post-training: 41%). The odds of cleanliness increased on average by 1.33 weekly during the pre-training period (CI = 1.11–1.60), and by 1.08 (CI = 1.03–1.13) during the post-training period. Cleaning action improved only in the pre-training period. Detection of *S. aureus* on hospital surfaces did not change substantially. The intervention was well received and considered feasible in this context. The major pitfalls in the implementation were the limited number of training sessions at the hospital level and the lack of supportive supervision. A systems barrier to implementation was lack of regular cleaning supplies.

**Conclusions:**

The evaluation suggests that improvements in microbiological cleanliness are possible using this intervention and can be sustained. Improved microbiological cleanliness is a key step on the pathway to infection prevention in hospitals. Future research should assess whether this bundle is cost-effective in reducing bacterial and viral transmission and infection using a rigorous study design.

## Background

Healthcare associated infections (HAI) are estimated to affect 15% of hospital inpatients in Tanzania and similar high rates are found in other low and middle-income countries (LMICs) [1, 2]. HAI are a major issue for patient safety with implications for patient morbidity and mortality and healthcare costs [1, 3]. The problem is heightened with recent epidemics such as COVID-19 and Ebola [4], and is compounded by the indiscriminate use of antibiotics [5, 6]. As more women deliver in healthcare facilities and undergo Caesarean-sections, the risk of HAI on maternity units in LMICs is increasingly concerning [7–9]. Newborns delivered in hospitals in LMICs are 3–20 times more likely to develop an infection compared to those in high-income countries [10]. A critical but often neglected aspect of HAI prevention is basic environmental hygiene, particularly surface cleaning and linen management [11, 12]. Systematic removal of microorganisms from hospital surfaces impedes direct bacterial and viral transmission to patients and indirect transmission via the hands of healthcare workers or medical equipment. Cleanliness also contributes to providing a respectful environment for women, babies and healthcare workers.

As in many low-income settings, environmental hygiene is poor in many Tanzanian hospitals [13, 14]. Over 80% of Tanzanian hospitals in 2015/16 did not meet basic cleaning indicators based on visual cleanliness despite almost all having disinfectant and running water available [15]. Our prior research in seven Tanzanian hospitals revealed frequent bacterial contamination of maternity beds, including with *Staphylococcus aureus—*a leading pathogen in terms of infection burden [16]. Furthermore, the formal training and support mechanisms for staff with environmental hygiene responsibilities are inadequate [12, 17].

TEACH CLEAN is an intervention aimed at improving environmental hygiene in maternity units in low-resource settings. It was created by the Soapbox Collaborative jointly with NHS Grampian (UK) based on international guidelines for environmental hygiene. It was pilot-tested in The Gambia in 2016, and used in India and Cameroon [18]. Key features include participatory methods and pictorial guidelines to facilitate learning for hospital cleaners, who typically have low education and literacy levels [12, 19, 20], TEACH CLEAN comprises the following stages:

1. Preparatory stage: engagement with hospital managers, selection of facility *cleaning champions* in each hospital; assessment of environmental hygiene status and resources; and adaptation of TEACH CLEAN to the local context;
2. Training stage: training of facility *cleaning champions* to educate and supervise existing staff with environmental hygiene responsibilities;
3. Supervision stage: ongoing mentorship of *cleaning champions* while they educate and supervise existing staff with environmental hygiene responsibilities.

Cleaning champions are selected on the basis of having a supervisory or senior role within the ward, prior understanding of infection prevention in relation to environmental hygiene, good communication skills and willingness to develop knowledge and take on the role of champion. The seven TEACH CLEAN training modules cover cleaning agents, frequency of cleaning, cleaning techniques, importance of environmental hygiene for HAI prevention, and, specifically for champions, techniques for supervising staff and methods for attracting sufficient resources. The TEACH CLEAN bundle is currently the only cleaning training for hospitals in resource-limited settings endorsed by several international guidelines including the recent “Best Practices for Environmental Cleaning in Healthcare Facilities: in resourced-limited settings” [11].

**Fig. 1:**
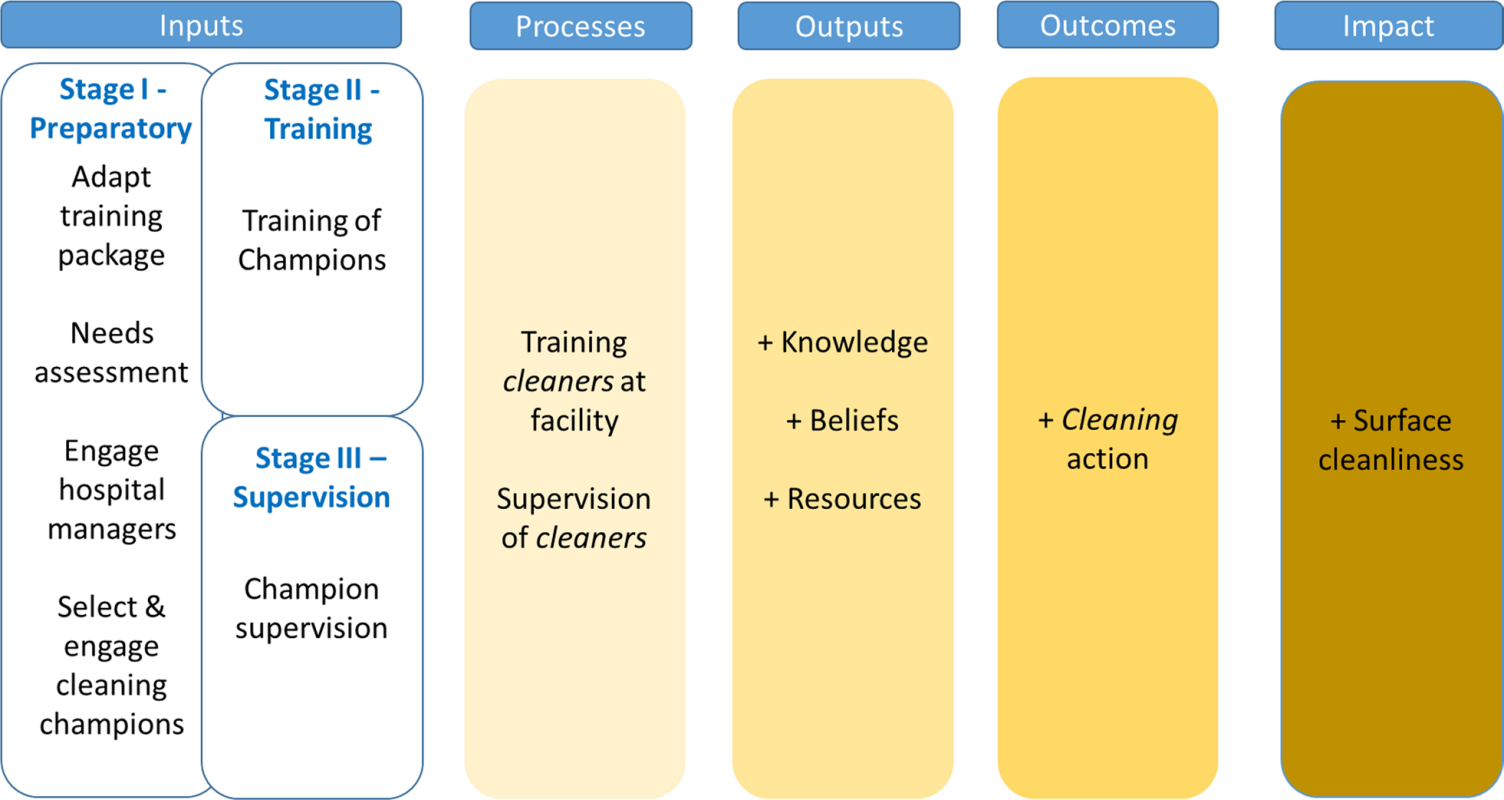
Simplified theory of change. Note: *Cleaners* stand for all staff with environmental hygiene responsibilities

In this paper, we report the results of a pragmatic pilot study to better understand the impact of the TEACH CLEAN intervention in a training-naïve, low-resource maternity and newborn ward setting. The TEACH CLEAN bundle was delivered by a local training institute in Tanzania, the Muhimbili University of Health and Allied Sciences (MUHAS), with technical support from The Soapbox Collaborative. We hypothesized that the intervention improves the training and supervision of staff with environmental hygiene responsibilities (e.g. cleaners, enrolled nurses, and laundry staff), increases knowledge about environmental hygiene and appropriate cleaning behaviour (including technique), ultimately, improves surface microbiological cleanliness (Fig. 1 and Additional file 1). To our knowledge, this is the first study to comprehensively evaluate an intervention on hospital environmental hygiene in a low-resource setting.

## Objectives

The study aimed to:

1. Assess the change in surface microbiological cleanliness
2. Assess the change in cleaning action frequency, knowledge and beliefs
3. Describe the intervention implementation—fidelity, adaptations, dose and reach
4. Describe the context in relation to resources and barriers to environmental hygiene

## Methods

### Setting and design

The CLEAN pilot study ran between April 2018 and July 2019 in three high-volume public hospital facilities in Dar es Salaam, Tanzania (average monthly deliveries: 1089–1393). The evaluation was conducted in four wards in each facility: labour ward, post-natal ward (vaginal deliveries), post-natal caesarean-section ward, neonatal ward (in 2 facilities) and kangaroo mother care (KMC) ward (in 1 facility). The project was a collaboration between the London School of Hygiene and Tropical Medicine (LSHTM), the Ifakara Health Institute (IHI) and the Soapbox Collaborative. MUHAS was contracted directly by IHI as a training institution.. MUHAS was contracted directly by IHI as a training institution.

**Fig. 2:**
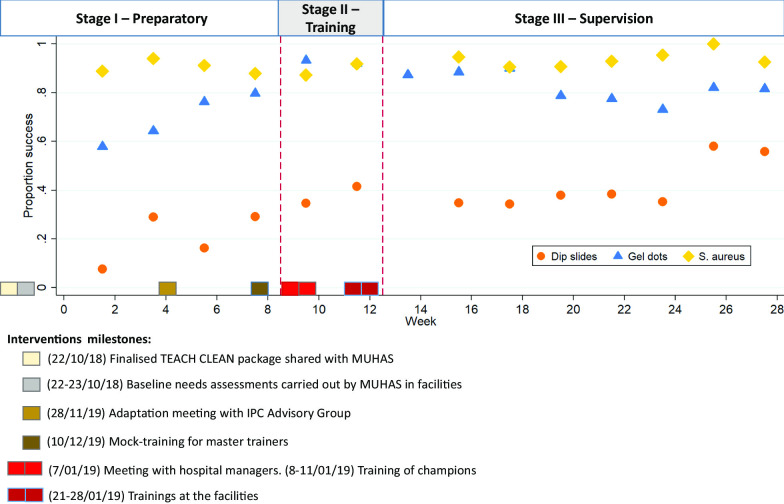
Graph showing the intervention milestones and the proportion of success for cleanliness (ACC < 2.5 cfu/cm^2^), absence of *S. aureus*, and cleaning action (gel dot removal)

Ten days of formative observation (August–September 2018) identified key environmental sites at which to measure cleanliness. Pre-training data collection ran for 8 weeks from 28th October 2018 (roughly coinciding with stage I of implementation). Training of champions (stage II) and subsequent training of cleaners at facilities took place from 7 to 28th January 2019 (weeks 9–12). Post-training data collection (intended to coincide with stage III) ran for 15 weeks from 29th January to 24th May 2019 (weeks 13–28). Figure 2 summarizes this timeline.

This study design prospectively evaluated the intervention as a whole, and offered a before-and-after comparison of impact of the main training (stage II), albeit with a baseline period (stage I) where some intervention activities were already taking place. We report the study according to SQUIRE guidelines for quality improvement studies in healthcare (Additional file 2) [21]. This study was intended to be a pragmatic pilot evaluation, reflecting the continuous nature of a quality improvement intervention.

### Data collection

#### Weekly quantitative data collection

Weekly data collection aimed to measure microbiological cleanliness (Objective 1), physical cleaning action (Objective 2) and contextual information (Objective 4).

*Sampling sites* The unit of measurement for cleaning action and microbiological cleanliness was key hand-touch sites—patient beds in our study. Formative observation identified patient beds as surfaces most frequently touched by healthcare workers, closest to the patient, thus providing the greatest risk of pathogen cross-transmission [22, 23], with some beds used more frequently than others (Additional file 3). Other studies report bed frames as key hand-touch sites in hospitals [11, 23]. We also sampled an equipment trolley, a bedside locker, a sink, and a water tap because these were also high-touch sites and beds were limited in one ward. Ten sites were selected in each of 11 wards and five sites in the KMC ward, giving a total of 115 sites. We aimed to collect 20 samples at each site, half during the pre-training and half during the post-training periods. In order to minimize staff behaviour change in response to the evaluation, we randomly varied the visit day and the physical sites sampled on each screening occasion.

*Dipslides* Dipslides are a widely used method for measuring surface microbiological cleanliness, in hospitals and elsewhere [24]. We used dipslides coated with a non-specific agar on one side for measuring total Aerobic Colony Counts (ACC/cm^2^) and a selective agar on the other (Baird-Parker agar, to determine the presence of *S. aureus*; Dimanco, UK). Both sides are applied consecutively to adjacent areas of the sampling site, immediately before assessment of the gel dot removal (see below). Dipslides were collected from two hospitals each week on a rotating basis. They were transported to the IHI laboratory on the day of collection and incubated in aerobic conditions for 14–24 h at 37 °C. Colonies were enumerated by visual inspection. Any potential *S. aureus* colonies (i.e. appropriate morphology and coagulase positive) were sub-cultured onto blood agar and re-incubated for a further 24 h at 37 °C in air, before repeat coagulase testing. For quality control, every 10th dipslide was also examined by an internal 2nd reader and every 20th dipslide was examined by an external 3^rd^ reader. Once we started the study, we found that if the samples were formally incubated for > 24 h, many of the colonies “over-grew” so became hard to enumerate. We found that a relatively short incubation (in comparison to what is described elsewhere) worked better—we assumed this was due to higher ambient temperatures and the interval from sample collection to receipt in the lab.

*Fluorescent gel dots* To measure physical cleaning, data collectors applied a transparent gel solution (gel dot) to chosen sites and subsequently (after 8–24 h) used an ultraviolet light to determine whether these had been substantially disrupted or totally removed [25–27]. Gel dots do not require any particular technique or pressure to remove; a wet wipe is sufficient. Data collectors used the EvaluClean software (copyright: GAMA Healthcare, UK) to record the exact position of the gel dot at each site (Additional file 4a). A layout for each ward helped the data collectors identify the correct location (Additional file 4b). Time of application was randomly allocated to either morning, afternoon or night shift. Gel dot removal was measured the next day, after general cleaning took place (between 8 and 10 AM) and recorded in EvaluClean software. Gel dot data were collected from all three hospitals each week.

*Observation checklist* During each visit (twice weekly per hospital) we collected information on the number of patients and availability of water and chlorine using a pre-coded observation checklist. Data collectors were asked to report any incidents they learned about informally, which may have affected resources for environmental hygiene.

*Training, piloting and data monitoring* Training of data collectors and piloting of dipslides, gel dots, and the pre-coded observation checklist ran for 6 weeks between September and October 2018 in 2 study wards. During the main study, data collectors met weekly with the research coordinator at IHI and every 2 weeks with the project coordinator at LSHTM. Data were uploaded and synced weekly and regular feedback provided.

#### Questionnaire

A structured questionnaire was administered to cleaning champions and other staff with environmental hygiene responsibilities, before the training (December 2018–January 2019), and afterwards (April–May 2019) to assess whether participants’ knowledge and beliefs around environmental hygiene changed as a result of the training (Objective 2). We used a capture-recapture sampling method to deliver this as it was unclear before the training who would be recruited as a training participant. Piloting of the pre-training questionnaire was undertaken with a convenience sample of 26 people in October 2018 in two non-intervention wards. Both pre- and post-training questionnaires contained identical questions except that the post-training questionnaire featured additional questions on the relationship between the respondent and the champions.

The constructs (e.g. enablement, social norms, experiential attitudes) were developed using the Behaviour Change Wheel [28] and the cross-culturally validated Integrated Behavioural Model [29]. Knowledge questions were based on the content of TEACH CLEAN. Questions on supervision, resources and support were influenced by the Australian REACH study questionnaire and our Theory of Change [27].

#### Observation

Observation, conducted by a qualitative researcher (YS), aimed to gather information about the implementation of the training intervention (Objective 3) and the context in which it was deployed (Objective 4). Five days of observation were conducted during the training of champions at MUHAS and six days during the training of staff in the three hospitals. An observation guide was used to capture information on training content, delivery methods, and relationship between trainers and trainees. The observer did not participate in any training delivery.

#### Qualitative interviews and focus group discussions

Focus group discussions (FGD) and one semi-structured in-depth interview were conducted to understand the training participants’ experience, the implementation of training and any barriers or facilitators to training success (Objective 3 and 4)—see Additional file 5 for a sample of the topic guide. Participants were purposively sampled. One of the MUHAS facilitators was interviewed and two FGDs conducted in each facility, one with champions and one with the staff they trained (in total 40 participants). The topic guide for the interview included questions around their experience in adapting TEACH CLEAN and any perceived similarities and differences between implementation in the three hospitals. The topic guide for FGDs included for example, topics around enablers and barriers to ensuring environmental hygiene in the facility, experience of the training, and other competing priorities with environmental tasks. Each discussion or interview lasted 60–90 min. All interviews occurred in each of the relevant hospital.

### Data management and analysis

#### Quantitative weekly data

Data were cleaned, checked for inconsistencies and analysed using Stata/MP v14.2 software. Variables were coded and analysed according to the definitions in Table 1. We used descriptive statistics to summarise and compare impact outcomes (cleanliness standards: ACC < 2.5 cfu/cm^2^ and absence of *S. aureus*) [30] and process outcomes (physical cleaning action performed, i.e. gel dots removed) during the pre-training and post-training periods. We determined the bi-weekly proportions of these outcomes and describe the time trends throughout the study. With the planned data collection measurement, we had over 90% power to detect a 10% increase in surface microbiological cleanliness, as defined below, from a 20% cleanliness baseline (Additional file 6).

**Table 1:** Variable definitions

Type	Variable	Definition
Impact	Cleanliness (Aerobic Colony Counts—ACC)	Binary (< 2.5 cfu/cm^2^ = clean; ≥ 2.5 cfu/cm^2^ = not clean) Categorical (0; > 0 to < 2.5; 2.5 to < 12; 12 to < 40; 40+)
Impact	Cleanliness (*S. aureus*)	Binary: presence or absence of *S. aureus*
Process outcome	Cleaning action (gel dots)	Binary (cleaning performed: gel dot completely removed; failure: gel dot not removed or only partially removed)
Outcome predictor	Ward type	Categorical (labour ward, postnatal vaginal deliveries, post-natal c-section deliveries, neonatal ward OR KMC ward)
Outcome predictor	Hospital	Categorical (1, 2, 3)
Outcome predictor	Bed occupancy	Occupancy is the number of patients divided by the number of beds in that specific ward [Note: Number of patients was collected during each visit. For labour ward only it refers to the number of patients present during last shift]
Study characteristic	Shift type	Categorical (gel dots applied either morning, afternoon or night)
Study characteristic	Bed frame/mattress	Binary (mattress or bed sampled)
Context	Water availability	Binary (whether water is flowing from at least one access point: yes, no)
Context	Chlorine availability	Binary (whether chlorine is available in the central store and within the expiry date: yes, no)

We used multivariable logistic regression (with random effects to account for clustering by sampling location) to estimate the weekly change in odds and confidence intervals (95%CI) for the impact and process outcomes. We adjusted for potential predictors of the outcome: ward type, hospital and bed occupancy. We conducted two sensitivity analyses for the main impact outcome (ACC pass/fail): (a) re-calculating bed occupancy at one facility excluding infrequently used delivery beds, and (b) restricting analyses to data collected from bedframes in case staff might engage with cleaning frames and mattresses differently. There were insufficient data to analyse mattresses separately.

We calculated the polychoric correlation coefficient between microbiological cleanliness (using dipslide ACC) and frequency of cleaning action (gel dots). We calculated the intra-cluster correlation separately for wards and hospitals to measure the relatedness of data [31]. Sensitivity, specificity, positive predictive value, and negative predictive value were calculated using conventional methods. Results from gel dots (“test”) were compared the dipslides ACC results (“reference”).

#### Questionnaire

After data cleaning and preliminary exploration, a number of items were excluded from further analysis. Perceived control scale and the experiential attitudes scales were unsuitable for analysis due to poor inter-item reliability (Cronbach’s alphas < 0.70). Three knowledge questions were not analysed because the locally adapted training did not match the response options we designed. Responses regarding interaction with cleaning champions in the post-training questionnaire were dropped as it emerged that there was a misunderstanding regarding who had been formally designated in this role; and who considered themselves some kind of informal cleaning ambassador. Norms questions were either at ceiling or relevant sample size was too small.

We analysed the data for those respondents who attended the training and who completed both questionnaires. We performed descriptive analyses using the questionnaire responses. We calculated the appropriate measure for each question (e.g. percentage in each answer category). For questions included in both questionnaires, we tested differences between dichotomous paired responses using McNemar’s test and changes to ordered categorical responses using the sign test method.

#### Qualitative

All FGDs and the interview were conducted in Swahili and recorded using a digital recorder. Fieldnotes were documented on daily basis during and immediately after the observations. Weekly debriefing sessions with a senior member of staff were held to discuss interviews and fieldnotes. The audio recordings were transcribed verbatim. One FGD and the interview were translated into English and independently coded. For the other interviews, only quotes included in the paper were translated into English. Transcripts were managed and coded using NVivo 12 Software. Analysis of the qualitative data occurred alongside data collection. We employed thematic analysis that involved (1) familiarization of data through re-listening to audio and/or re-reading of transcripts and observation fieldnotes; (2) initial coding; (3) searching for themes; (4) reviewing themes; (5) defining and naming themes; (6) and writing up the report [32].

We report the qualitative findings on the implementation following MRC guidelines for process evaluation around the following themes: adaptation, fidelity, dose and reach, and context [33].

#### Ethics

The study received ethics approval from LSHTM, IHI and the National Institute for Medical Research, Tanzania.

During the formative phase for the selection of hand-touch sites, we collected written consent from all female patient and birth attendants present during the observation period. Women were informed that the person being observed was the birth attendant and that no information would be collected on them. Written consent from healthcare workers was gathered during the questionnaire, the FGDs and the in-depth interview. We obtained oral consent from healthcare workers for the data collection on the environment and the context, and during observation of training of the champions and transfer training in the hospitals.

#### Data sharing

Anonymized quantitative data for the main impact and process outcomes is deposited on the LSHTM Data Compass (https://doi.org/10.17037/DATA.00001937). Individual questionnaire information and qualitative findings have not been made available due to the small sample size that might compromise anonymization. Some of this data can be requested from the corresponding author.

## Results

### Impact and process outcomes

#### Microbiological cleanliness (ACC and *S. aureus*)

In total, we collected 1200 dipslides for ACC evaluation of which 10 (0.8%) we could not have a clear reading of the ACC category and therefore these were not used in the analysis. The final dataset includes 1190 data points. On each data collection day, we gathered between 20–34 samples (mean = 26.4, SD = 3.3). Only 1.3% of datapoints were missing when compared against the collection schedule. The proportion of cleanliness (ACC < 2.5 cfu/cm^2^) was 19.1% during the pre-training period and 40.7% during the post-training period (Table 2). From visual inspection of the data, improvement began during the pre-training period and continued during the study period (Fig. 2).

**Table 2:** Impact and process outcomes by study characteristics and outcome predictors

	Microbiologically clean (ACC < 2.5 cfu/cm^3^) n/N (%)	Absence of *S. aureus* n/N (%)	Cleaning action performed (gel dots removed) n/N (%)
	N = 1190	N = 1205	N = 2238
Overall	404/1190 (34.0)	1125/1205 (93.4)	1796/2238 (80.3)
Time period			
Pre-training	69/361 (19.1)	340/367 (92.6)	449/647 (69.4)
Training	63/161 (39.1)	150/165 (90.9)	314/339 (92.6)
Post-training	272/668 (40.7)	635/673 (94.4)	1033/1252 (82.5)
Missing	0	0	0
Hospital			
1	131/436 (30.1)	412/441 (93.4)	658/767 (85.8)
2	121/343 (35.3)	323/347 (93.1)	516/668 (77.3)
3	152/411 (37.0)	390/417 (93.5)	622/803 (77.5)
Missing	0	0	0
Ward type			
Postnatal c-section	116/304 (38.2)	287/308 (93.2)	486/587 (82.8)
Postnatal vaginal	117/307 (38.1)	287/309 (92.9)	462/579 (79.8)
Labour	97/307 (31.6)	297/312 (95.2)	487/583 (83.5)
Neonatal/Kangaroo	74/272 (27.2)	254/276 (92.0)	361/489 (73.8)
Missing	0	0	0
Audit location			
Bedframe	315/830 (38.0)	784/839 (93.4)	1216/1555 (78.2)
Mattress	83/313 (26.5)	297/317 (93.7)	532/601 (88.5)
Missing location	6/47 (12.8)	44/49 (89.8)	48/82 (58.5)
Shift type			
Morning	144/438 (32.9)	401/441 (90.9)	706/833 (84.8)
Afternoon	153/456 (33.6)	431/461 (93.5)	594/807 (73.6)
Night	107/296 (36.2)	293/303 (96.7)	496/598 (82.9)
Missing shift	0	0	0
Day of week			
Sunday	–	–	–
Monday	178/579 (30.7)	535/587 (91.1)	608/738 (82.4)
Tuesday	–	–	–
Wednesday	226/611 (37.0)	590/618 (95.5)	571/737 (77.5)
Thursday	–	–	–
Friday	–	–	617/763 (80.9)
Missing day	0	0	0
Bed occupancy			
Low (< 0.75)	99/285 (34.7)	259/288 (89.9)	474/582 (81.4)
Medium (0.75–1.25)	156/477 (32.7)	452/483 (93.6)	691/854 (80.9)
High (> 1.25)	137/388 (35.3)	376/394 (95.4)	537/697 (77.0)
Missing occupancy	12/40 (30.0)	38/40 (95.0)	94/105 (89.5)

After accounting for the predictors of the outcome (ward type, hospital and bed occupancy), the odds of cleanliness increased on average by 1.33 each week during the pre-training period (CI = 1.11–1.60), and by 1.08 (CI = 1.03–1.13) during the post-training period (Table 3). The results from the additional sensitivity analyses are consistent with our main results (Additional file 7). The ICC for wards was 0.03 (CI = 0.00–0.06) and the ICC for hospital was < 0.01 (CI = 0.00–0.02).

**Table 3:** Weekly change in odds of outcome success for the pre-training (weeks 1–8) and post-training (weeks 13–28) periods

	n	Crude odds ratio (CI)^§^	*p* value	N	Adjusted odds ratio (CI)*^§^	*p* value
Microbiological cleanliness (ACC < 2.5 cfu/cm^2^)						
Pre-intervention	361	1.22 (1.04–1.44)	0.014	355	1.33 (1.11–1.60)	0.002
Post-intervention	668	1.08 (1.03–1.12)	< 0.001	652	1.08 (1.03–1.13)	< 0.001
Absence of *S. aureus*						
Pre-intervention	367	1.03 (0.85–1.26)	0.731	361	1.12 (0.87–1.45)	0.379
Post-intervention	673	1.04 (0.96–1.13)	0.350	657	1.02 (0.93–1.11)	0.734
Cleaning action (gel dots)						
Pre-intervention	647	1.23 (1.13–1.33)	< 0.001	634	1.25 (1.15–1.36)	< 0.001
Post-intervention	1252	0.95 (0.92–0.98)	0.002	1190	0.97 (0.94–1.01)	0.098

^*^Controlled for hospital, ward type, and bed occupancy

^§^Accounts for site level clustering

We also investigated how the different categories of ACC cleanliness changed from before to after the training (See Additional file 8). We did not investigate these accounting for time and potential predictors of the outcome, as our sample size was limited for some of these categories. The number of dipslides showing the worst growth category (40 + CFU) was substantially lower during the post-training period at 2.1% compared with 15.9% during the pre-training period.

We collected 1205 data points on presence or absence of *S. aureus*. Absence of *S. aureus* was at 92.6% during the pre-training period and 94.4% during the post-training period, suggesting little change across the study period. There was no evidence that recovery rate of *S. aureus* changed over time, after accounting for potential predictors of the outcome (Table 3).

#### Cleaning action (gel dots)

We collected 2,238 data points on cleaning action: each data collection day, we audited between 18 and 36 gel dots (mean = 27.3; SD = 3.3). The proportion of successful cleaning action was 69.4% during the pre-training period, and 82.5% during the post-training period (Table 1). Cleaning action appears to improve over the course of the pre-intervention period (OR = 1.25; CI = 1.15–1.35), reaching a peak during the training period followed by a levelling off or slight decline in the post intervention period (OR = 0.97; CI = 0.94–1.01) (Fig. 2, Table 3). The ICC for wards was 0.03 (CI = 0.00–0.06) and the ICC for hospital was 0.01 (CI = 0.00–0.04).

**Fig. 3:**
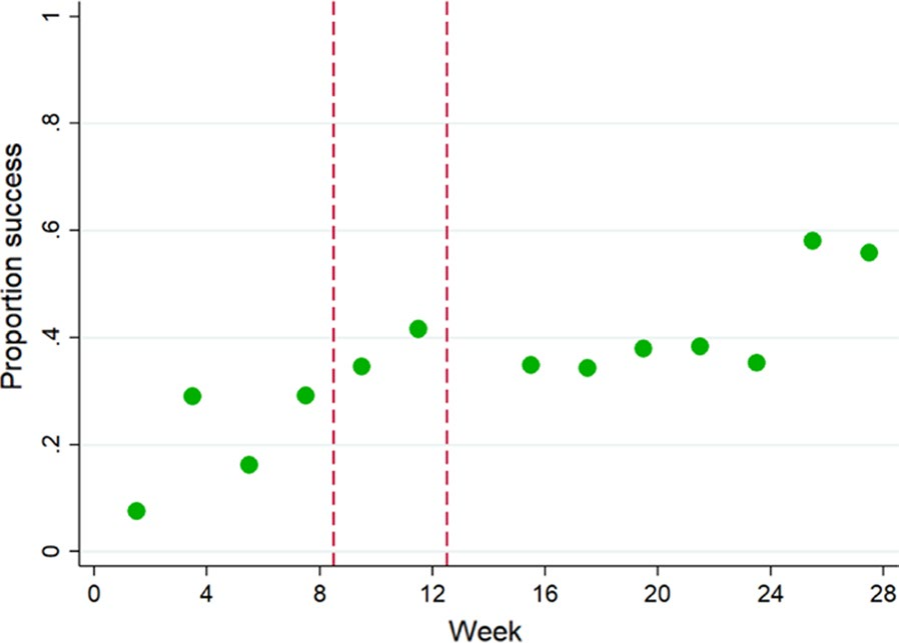
Proportion microbiologically clean (ACC < 2.5) if gel dot successfully removed, by week. Note: red lines define the training period

Figure 3 shows the rate of cleanliness success (ACC < 2.5 cfu/cm^2^) when restricted to the paired samples where the paired gel dot was removed i.e. cleaning action occurred. This continuous upward trend indicates that as the study progressed, if gel dots were removed then there was lower probability of significant microbial contamination being detected.

#### Correlation, sensitivity and specificity between dipslides and gel dots

There was no correlation between the gel dot and dipslide results, with an overall correlation coefficient of 0.06. The lack of correlation held across all study characteristics we tested including intervention periods (details on specificity, sensitivity and predictive values are in Additional file 9).

Other outcomes were collected but not presented here as they were not part of our initial theory of change. Improvements in appropriate storage of sharp disposal boxes and mops are shown descriptively in online file (https://doi.org/10.17037/DATA.00001937).

### Knowledge and beliefs

Nineteen female hospital staff who participated in the TEACH CLEAN training and who responded to both questionnaires were included in the analysis. Four of these were champions. This sample included cleaners, registered nurses, and medical attendants. Only four (21%) had received any previous training on cleaning procedures. 79% had worked in the maternity or newborn wards for more than one year. Two-thirds of the interviewed staff worked exclusively in these wards (68%) and also supervised other staff with cleaning responsibilities (63%).

The proportion of respondents answering knowledge questions correctly improved substantially for all three questions (Table 4). However, none of the three questions relating to resource availability for cleaning indicated a change in perceived availability.

**Table 4:** Knowledge and beliefs questions

	Pre-training	Post-training		*p* value
	# (%)	# (%)		
Questions with correct or incorrect answers^1^				
Whether respondent mentioned “unhygienic surfaces on the ward” as possible cause of umbilical cord infection	3/19 (16%)	12/19 (63%)		< 0.001
Correctly answered “what is best to add to water before routine daily cleaning of floors and walls”	2/19 (11%)	7/19 (37%)		0.059
Correctly answered “when should you empty the sharps bin”	11/19 (58%)	17/19 (89%)		0.034

	Mean (95% CI)	Mean (95% CI)		*p* value
Questions with scales of 1–5 (1 = very difficult/strongly disagree; 5 = very easy/strongly agree)^2^				
Availability of resources for cleaning				
How difficult or easy is it to get all the equipment you need for cleaning?	4.1 (3.6–4.6)	3.9 (3.2–4.6)		0.788
How easy/difficult is it to complete your cleaning tasks in the time available?	3.1 (2.3–3.8)	3.6 (2.9–4.3)		0.424
If there is not enough chlorine solution, I tell my supervisor and it is supplied	4.8 (4.6–5.0)	4.8 (4.7–5.0)		0.812

*N = 19 respondents

^1^*p* values for these questions were calculated using McNemar’s test

^2^*p* values for these questions were calculated using the sign rank test

### Implementation

#### Adaptation

The initial needs assessment was carried out by MUHAS, with data analysis conducted by the Soapbox Collaborative. A supervisory committee of local stakeholders specialized in infection prevention and control met at least once to advise on the adaptation of TEACH CLEAN and were presented the needs assessment results. Adaptation included revision of the training based on the Tanzanian National IPC Guidelines [34]: one small difference was identified around the mops storage which caused some difficulties for the champions when training their peers.

The timing and content of the adaptation was not optimal according to the Soapbox Collaborative’s recommendations. For example, the Swahili translation of the illustrated guidelines was only received the day before the training of champions, leaving no time for back translation. In addition, MUHAS did not follow through with two of their own recommendations for the adaptation stemming from the needs assessment, i.e. new local photos capturing the local contexts were not included in the training material and discussion prompts were not translated into Swahili.

#### Fidelity

The selection of champions was based on the findings from the needs assessment and was considered appropriate by the local stakeholders and the hospital staff who were trained by the champions. Cleaners no longer address champions as "sister", they address them positively as "teacher". Champions were a mix of registered and enrolled nurses, assistant nurse officers and environmental health officers (Additional file 10). The training of champions took place from 8 to 11th January 2019, followed by four days of training at each facility between 21st and 28th January. All technical modules were delivered with similar content, timeframe and order as suggested by TEACH CLEAN, except for the modules on Supportive Supervision and Quality Improvement that were excluded from the Tanzanian Facilitators Guide; the module on housekeeping (including cleaning) which was taught last in one hospital; and the practical exercises that were all taught during the last day at one hospital instead of being interspersed with the module theory. Participatory techniques were used in all training sessions and were well received by all participants interviewed.

“…the second thing I liked [during the training], with the adult participatory technique, those people [champions] felt like they owned the training….there were no slides, it was not a lecturing training, it was hands on, more participation, often participants were talking more than facilitators, so I liked the method” ~ In-depth interview, IDI01_MUHAS

Participants commented on inadequate space for the training in two facilities and delays in receiving necessary equipment for training. Supportive supervision from MUHAS to the champions beyond the training at the facility did not occur—meaning that the whole of stage III was not implemented (Fig. 1). However, a WhatsApp group was set by MUHAS to support the champions in delivering the facility-level training.

#### Dose and reach

Eighteen champions and 45 staff with environmental hygiene responsibilities were trained across the three hospitals—16 in each of the two larger hospitals and 13 in the third. These 45 staff were a mix of medical attendants (18), cleaners (22), enrolled nurses (2) and laundry staff (3). Many staff with environmental hygiene responsibilities did not receive training. In the in-depth interviews and in follow up conversations with staff the qualitative researcher was told of problems with staff trying to implement new cleaning practices were challenged by other staff who had not had the training which at times caused tensions.

“… one off transfer training is not enough because there are many cleaners who need to be trained…”. ~ In-depth interview, IDI01_Training College, MUHAS

Preparation for the training by the champions was different across the three hospitals. In addition, the training in one facility did not include the pre- and post- knowledge test included in TEACH CLEAN.

### Context

Water was nearly always available (98% of the time) during the study period. Chlorine was always available when audited. However, in the in-depth interviews, a lack of supplies for cleaning, waste segregation and linen handling was perceived to be hindering the success of the intervention.

“We are trying but we have not reached the goal because of lack of availability of equipment such as mops… [For example], we were told to use mops in the morning and dry them up and use another mop in the afternoon. This has not been feasible because we do not have [them]… if there is one mop we go around [using] it” ~ FGD facility2, cleaners, P7

One important element of the context was the use of privately contracted cleaning services. From the questionnaire, about half (55%) of the respondents were contracted by a private company, whilst the others were hospital staff. However, this seemed a manageable issue because during implementation these cleaners were trained and supervised by the selected champions.

From the observation, we are aware that during the training, cleaning champions and staff with environmental hygiene responsibilities knew about the presence of our evaluation. With regards to other concurrent interventions, we learned the Tanzanian Ministry of Health was also rolling out a new quality improvement monitoring system that includes an indicator on visual cleanliness [35].

Finally beyond TEACH CLEAN, 80% of staff who responded to the questionnaire (n = 73) had not received any formal training in environmental hygiene training; of these 55% had no formal training at all; and 25% only received on the job training.

## Discussion

We conducted a pilot evaluation of the environmental hygiene intervention TEACH CLEAN in three high volume maternity and newborn units in hospitals in Dar es Salaam, Tanzania. To our knowledge, this is the first study in a low-resource setting that rigorously evaluates the impact from an environmental hygiene intervention on hospital cleaning. All impact and process outcomes improved during the study period except for *S. aureus* contamination.

Microbiological cleanliness (ACC < 2.5 cfu/cm^2^) increased during the study suggesting a positive effect of the intervention on cleaning quality. Cleanliness continued improving, albeit slowly, after the training took place, despite fluctuations in cleaning frequency. This may be the result of better cleaning techniques learned during the training. The improvement in staff knowledge following training also provides circumstantial but convergent evidence for this conclusion. We did not find evidence that *S. aureus* decreased on surfaces—an alternative marker of successful cleaning—during the study period. This may be explained by our limited sample size for this relatively rare outcome (average recovery across whole study = 7%). *Staphylococcus aureus* accounts for a quarter of clinical microbiological cultures among African newborns with a HAI—the single most common pathogen [36]. Future studies should be properly powered to detect changes in environmental contamination with this important indicator pathogen.

Cleaning action (measured by gel dots) improved during the pre-training period, but there was no statistical evidence for either improvement or decline post-training. Despite our efforts to conceal details of data collection and the focus of the study, ward staff might have been motivated by the regular presence of data collectors to increase their cleaning. This ‘Hawthorne-like effect’ can decline over time because of staff habituation, and may explain why improvements in cleaning action ceased by the end of the study. The pre-training period was one third shorter than intended which hindered our ability to capture a stable baseline.

Microbiological cleanliness was 19% for selected hand-touch sites during the pre-training period, and 41% during the post-training period – these are low against comparable estimates from high-income countries. In a teaching hospital in the United States, Boyce et al. found, using the same definition, that 77% of surfaces were clean (ACC < 2.5 cfu) [25]. Poor microbiological cleanliness (Mean ACC = 39 cfu during baseline) was also reported from paediatric units in South Africa [20]. However, the rate of success for cleaning action, i.e. removal of pre-placed invisible gel dots, was 69% during the pre-training period, and 83% during the post-training period. This was similar to, or higher than, levels reported from the US, Australia and South Africa respectively at 67%, 64–86% and 22–61% [20, 25, 27]. However, most of these studies assessed terminal cleaning (after patient discharge) rather than daily cleaning that we measured in our study suggesting – terminal cleaning is usually lower than daily cleaning. Our interpretation of these comparisons is that although cleaning is frequently attempted, the technique and resources are suboptimal in these Tanzanian units resulting in high levels of bacterial contamination. Indeed, in most low-resource hospital settings, no formal training of people with environmental hygiene responsibilities, in particular cleaning, is routinely performed [11, 12].

While the intervention was mainly delivered as intended in terms of activities, content and teaching methods, there were two major shortcomings. Firstly, only one round of the training was carried out in each facility reaching a limited number of staff. Secondly, supportive supervision was not delivered as expected by the training institute to champions after the facility-level trainings. This meant that champions were not then able to use the competency-based assessment and performance management techniques included in TEACH CLEAN. As expected given the “training-of-trainers” nature of the intervention, there was some degree of heterogeneity of implementation at the facility level.

During results dissemination, Tanzanian stakeholders at all levels (hospital, regional and national) expressed enthusiasm for incorporating TEACH CLEAN into routine training for hospital staff with environmental hygiene responsibilities. We hope that strong emphasis will be given to the importance of including modules on supportive supervision and quality improvement in the future. Furthermore, implementers should consider adding tools and strategies to support the availability of cleaning equipment in the hospitals. Insufficient equipment was identified by study participants as a barrier to successful implementation and perceptions of availability did not change during the study period. A budget planning tool could support champions to advocate for cleaning supplies with hospital management.

New international guidelines and local studies acknowledge that environmental hygiene is a key area for further research and implementation efforts [11, 37]. A training-of-trainers model is a relatively cheap and potentially sustainable way to deliver training to a large number of staff – in our study, the intervention activities cost about US$9000 to train 63 staff during four training sessions. The only randomized trial of a similar environmental hygiene bundle was recently conducted in Australia (the REACH study) and demonstrated an improvement in cleaning behaviour and a reduction in one type of HAI [27]. HAIs are an enormous problem in hospitals in LMICs. A randomized trial to investigate the effects of a trainer-of-trainer intervention for staff with environmental hygiene responsibilities in this context could be highly informative. In addition, more emphasis on environmental hygiene should be included in pre-service training.

Our evaluation of the intervention has two main strengths: use of objective measurements of both cleanliness and cleaning action, and a strong multi-disciplinary process evaluation. We used data collection methods rarely used in low-income hospitals: gel dots and dipslides [20]. Compared with other ways of measuring microbiological cleanliness, dipslides are relatively easy to use and analyse—this was key in a context of limited microbiological laboratory capacity. Florescent gel dots were also easy to use and allowed low-cost covert monitoring of physical cleaning action. The location where gel dots and dipslides were applied was randomly assigned to attempt concealment from hospital staff. Future studies using these methods should consider carrying out data collection less frequently than once a week to help reduce potential Hawthorne-like effects on staff behaviour. Additionally, gel dots could be used to support quality improvement efforts in this area and support the champions in supervising their fellow colleagues, as currently used in the UK and other high-income countries [38]. The second major strength of our evaluation is the variety of qualitative and quantitative information collected to help us understand why and how the intervention worked. We collected information on mechanisms of change such as knowledge, implementation fidelity, dose and reach as well as the context within which the intervention took place. This is essential for a thorough evaluation of a complex intervention [33, 39].

The main weakness in our evaluation of the intervention is that the baseline evaluation was run concomitantly with the start of the implementation (preparatory stage). Therefore, we are not able to conclusively say if the trends uncovered are the result of pre-existing trends in improvement, some other existing intervention, or the preliminary stages of the intervention; however, we believe the latter is the most plausible scenario. A second weakness is that the early start of implementation also meant that hospital staff were alerted to the upcoming training, potentially compromising the integrity of the evaluation despite our best efforts to keep study methods concealed. Additionally, the qualitative work suggested that champions and cleaners were alerted to the details of some evaluation methods, potentially further undermining the evaluation integrity. All efforts were made to show that the qualitative researcher was independent, but champions assumed that she was a training facilitator potentially influencing some responses. A third weakness is that we could not rigorously test the mechanisms of change quantitatively. Many of the questions in the questionnaire did not lend themselves to create scales as intended. The Australian REACH study also found the use of a beliefs questionnaire challenging, even when the questions modality (Likert-like questions) were based on validated questions. Whilst these are important shortcomings, this study was intended as a pilot evaluation of feasibility, rather than attempting to provide definitive answers on the intervention impacts.

## Conclusions

The TEACH CLEAN environmental hygiene intervention was feasible and well received in three large hospitals in Dar es Salaam, Tanzania. The evaluation suggests that it led to improvements in microbiological cleanliness which could be considerable and sustained. Although the teaching component of the intervention did not increase the physical cleaning action, there was some evidence that cleaning technique improved. Improved microbiological cleanliness is a key step on the pathway to infection prevention in hospitals [40]. Future research should assess whether this bundle is cost-effective in reducing bacterial and viral transmission and infection using a rigorous study design.

## Supplementary Information

**Additional file 1** “ToC and Assumptions". Details of the Theory of Change.

**Additional file 2** “SQUIRE guidelines”. SQUIRE checklist.

**Additional file 3** “Key hand-touch sites”. Selection of hand-touch sites.

**Additional file 4** “Sample size”. Sample size calculations.

**Additional file 5** “Tablet façade”. Visual of data collection tool.

**Additional file 6** “Layout”. Example of ward layout for data collection.

**Additional file 7** “Sensitivity analyses”. Sensitivity analyses results for microbiological cleanliness.

**Additional file 8** “ACC breakdown”. Results by categories of ACC.

**Additional file 9** “Sensitivity and Specificity”. Sensitivity and specificity calculations.

**Additional file 10** “Champions breakdown”. Details of champions selected.

## Data Availability

Anonymized quantitative data for main impact and process outcome is deposited on LSHTM website (https://doi.org/10.17037/DATA.00001937). Individual questionnaire information and qualitative findings, is not made available due to the small sample that might compromise anonymization. Some of this data can be requested from the corresponding author.

## Abbreviations

ACC: Aerobic Colony Counts
CFU: Colony Forming Units
FGD: Focus group discussion
HAI: Healthcare associated infections
IHI: Ifakara Health Institute
ICC: Intracluster correlation
KMC: Kangaroo Mother Care
LMICs: Low and middle-income countries
LSHTM: London School of Hygiene and Tropical Medicine
MRSA: Methicillin-resistant* Staphylococcus aureus*
MUHAS: Muhambili University of Health and Allied Science
