# Supplementary material for: The Clean pilot study: evaluation of an environmental hygiene intervention bundle in three Tanzanian hospitals
Source: Antimicrob Resist Infect Control. 2021 Jan 7;10:8. doi: 10.1186/s13756-020-00866-8 (PMC7789081; doi:10.1186/s13756-020-00866-8)
Supplement: Supplementary file 1 — Additional file 1 “ToC and Assumptions". Details of the Theory of Change. [file 13756_2020_866_MOESM1_ESM.docx]

# Additional File I – Theory of Change and assumptions


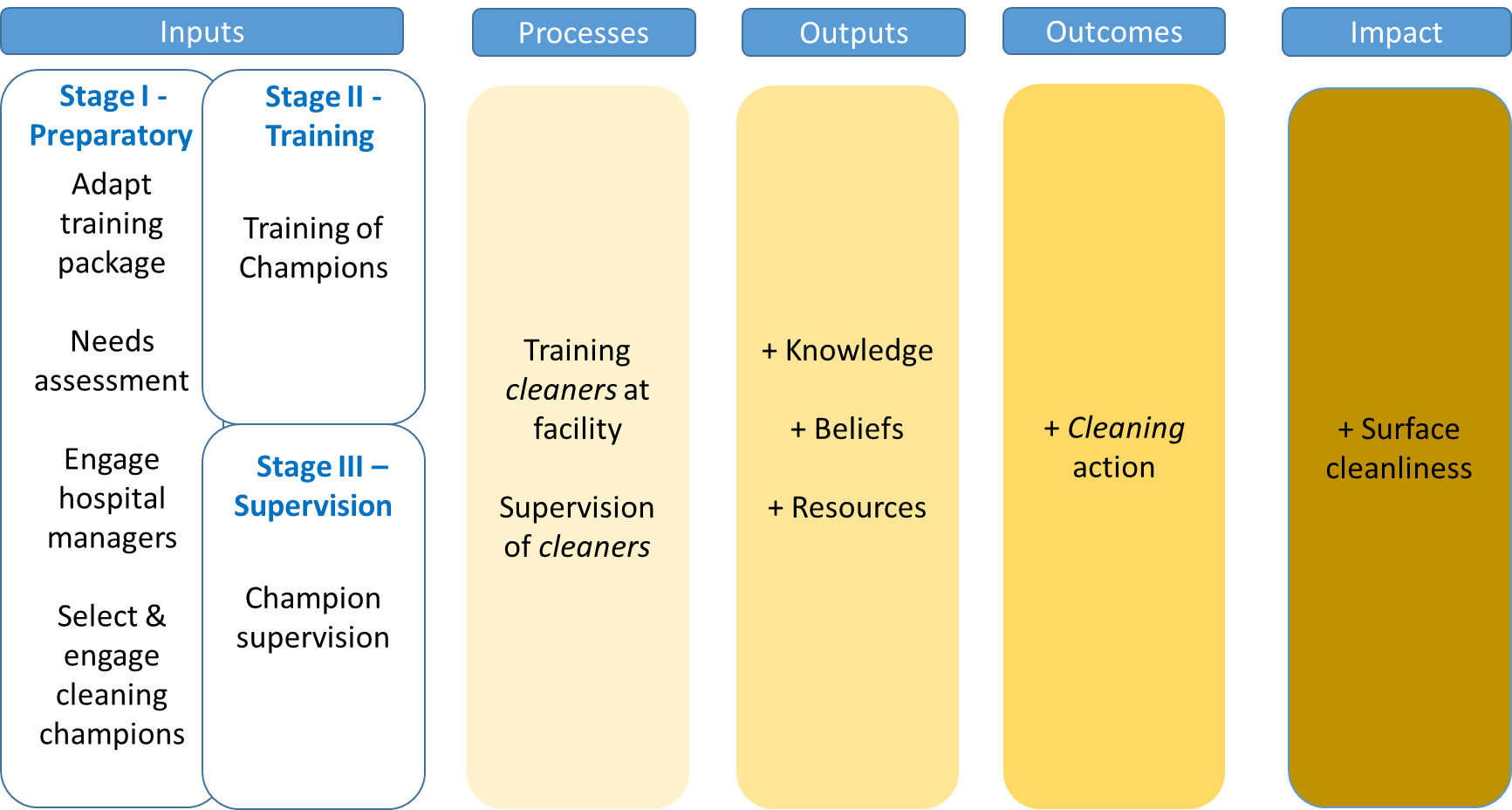


| **Assumption** | **Tool** | **Detail** |
| --- | --- | --- |
| 1.Facilities are not as clean as they should because cleaning is not done appropriately | Dipslides & Dots tool | Microbiology from the dipslides during baselines will tell us if facilities are sufficiently clean. Gel dots to be used to verify whether cleaning practices are performed appropriately |
| 2.Those who clean lack the knowledge and beliefs to carry out cleaning | Questionnaire | Questionnaire will assess knowledge and beliefs at baseline |
| 3. Resources are available for cleaning to take place | Observation and  Qualitative interviews | With observation we will collect information on: presence of key equipment needed for cleaning (water, chlorine ); rota per “patient volume”  Qualitative interviews we will investigate what are the gaps in the current system for environmental hygiene. |
| 4.TEACH CLEAN package is effective | Dipslides & Dots tool & Questionnaire | Using these tools we will check if cleaning, microbiological cleanliness and knowledge and beliefs have increased after the training |
| 5. Participatory/Adult learning techniques work well in delivering training objectives | Questionnaire  Qualitative methods (observation & interviews) | Questionnaire will tell us if professional identity has increased among champions and cleaners (whether they feel more empowered), if participatory training was well received  Qualitative observation will tell us if participatory/adult learning techniques were used during training; interviews with champions and training institute will tell us whether interviewees think these techniques were useful |
| 6. Appropriate champions are selected & hospital managers bought into the idea and follow champion selection guidance | Qualitative methods (interviews) | Qualitative methods will tell us whether the training institute and the hospital managers have used the guidance on champions’ selection to select the champions; and if the selection will be appropriate |
| 7.Training institute adapts training package appropriately | Qualitative methods | Observation during training (ToT and at the facility) and interviews with training institute will unpack the extent to which fidelity of training package was achieved |
| 8.Training changes Knowledge and beliefs | Questionnaire | Questionnaire will tell us if knowledge and beliefs have changed after the training |
| 9. Change in knowledge and beliefs will be sufficient to bring about changes in behaviour and ultimately cleanliness | Questionnaire and dots tool&dipslides | From the dots tool/dipslides we will be able to tell whether appropriate cleaning has increased.  Currently we will be able to tell what is the status of use/process around detergent solutions (questionnaire) and appropriateness of cleaning equipment storage (observation) |
| 10. the quality improvement module is sufficient to empower champions to ensure environmental hygiene lies within existing facility improvement initiatives, including whether enough resources (time and equipment) are available | Qualitative interviews | Interviews with champions, hospital managers, and the training institute (who provides supervision to champions) will tell us whether champions feel confident in asking for resources at higher managerial levels and whether environmental hygiene is placed within other quality improvement initiatives – whether it has been placed higher in the hospital agenda compared to prior the training |
| 11. Staff do not rotate much | Qualitative interviews  Questionnaire | Interviews with managers and champions should tell us whether major staff changes have occurred  Questionnaire will enquire about how long respondent served in that ward and whether they received the training |
| 12. In spite of potential contextual changes (Managerial changes, other major hospital initiatives) the training will be successful | Qualitative methods | Interviews with champions and managers will tell us about major contextual changes occurring during the study period. |
| 13. Champions have the time to train and supervise the ward attendants | Qualitative methods  Questionnaire | Quali interviews with champions and checking champions supervision notes will tell whether champions have enough time to train and supervise cleaners  Questionnaire will tell us whether supportive supervision has happened |
