# Supplementary material for: The Clean pilot study: evaluation of an environmental hygiene intervention bundle in three Tanzanian hospitals
Source: Antimicrob Resist Infect Control. 2021 Jan 7;10:8. doi: 10.1186/s13756-020-00866-8 (PMC7789081; doi:10.1186/s13756-020-00866-8)
Supplement: Supplementary file 2 — Additional file 2 “SQUIRE guidelines”. SQUIRE checklist. [file 13756_2020_866_MOESM2_ESM.docx]

# Additional File II – SQUIRE guidelines checklist

Link to SQUIRE guidelines: <http://squire-statement.org/index.cfm?fuseaction=Page.ViewPage&PageID=471>

| **Text Section and Item Name** | **Section or Item Description** | **Section in manuscript** |
| --- | --- | --- |
| **1. Title** | Indicate that the manuscript concerns an initiative to improve healthcare (broadly defined to include the quality, safety, effectiveness, patient- centeredness, timeliness, cost, efficiency, and equity of healthcare) | Yes |
| **2. Abstract** | 1. Provide adequate information to aid in searching and indexing 2. Summarize all key information from various sections of the text using   the abstract format of the intended publication or a structured summary such as: background, local problem, methods, interventions, results, conclusions | Yes |
| **Introduction** |  |  |
| **3. Problem Description** | Nature and significance of the local problem | Paragraph 1 and 2 |
| **4. Available knowledge** | Summary of what is currently known about the problem, including relevant previous studies | Paragraph 1 and 2 |
| **4. Available knowledge** | Informal or formal frameworks, models, concepts, and/or theories used to explain the problem, any reasons or assumptions that were used to develop the intervention(s), and reasons why the intervention(s) was expected to work | Paragraph 3 and 4 |
| **6. Specific aims** | Purpose of the project and of this report | Paragraph 5 and 6 |
| **Methods** |  |  |
| **7. Context** | Contextual elements considered important at the outset of introducing the intervention(s) | Paragraph 2 and 4 of the introduction |
| **8. Intervention(s)** | 1. Description of the intervention(s) in sufficient detail that others could reproduce it 2. Specifics of the team involved in the work | Paragraph 3-5 of the introduction |
| **9. Study of the Intervention(s)** | 1. Approach chosen for assessing the impact of the intervention(s) 2. Approach used to establish whether the observed outcomes were due to the intervention(s) | Paragraph 1-3 of the methods section |
| **10. Measures** | 1. Measures chosen for studying processes and outcomes of the intervention(s), including rationale for choosing them, their operational definitions, and their validity and reliability 2. Description of the approach to the ongoing assessment of contextual elements that contributed to the success, failure, efficiency, and cost 3. Methods employed for assessing completeness and accuracy of data | All paragraphs under the “data collection” section of the methods |
| **11. Analysis** | 1. Qualitative and quantitative methods used to draw inferences from the data 2. Methods for understanding variation within the data, including the effects of time as a variable | All paragraphs under the “data management and analysis” section of the methods |
| **12. Ethical Considerations** | Ethical aspects of implementing and studying the intervention(s) and how they were addressed, including, but not limited to, formal ethics review and potential conflict(s) of interest | Both paragraphs under the “ethics” section of the methods |
| **Results** |  |  |
| **13. Results** | 1. Initial steps of the intervention(s) and their evolution over time (*e.g.*, time-line diagram, flow chart, or table), including modifications made to the intervention during the project 2. Details of the process measures and outcome 3. Contextual elements that interacted with the intervention(s) 4. Observed associations between outcomes, interventions, and relevant contextual elements   e. Unintended consequences such as unexpected benefits, problems, failures, or costs associated with the intervention(s).  f. Details about missing data | 1. All paragraphs in the “adaptation” section under results 2. All paragraphs in the “impact and process outcomes”   section under results   1. All paragraphs in the “context” section under results 2. Not done quantitatively 3. Throughout the “implementation” section of the results 4. Throughout the impact and process outcomes”   section under results |
| **Discussion** |  |  |
| **14. Summary** | 1. Key findings, including relevance to the rationale and specific aims 2. Particular strengths of the project | Paragraph 1 in the discussion |
| **15. Interpretation** | 1. Nature of the association between the intervention(s) and the outcomes 2. Comparison of results with findings from other publications 3. Impact of the project on people and systems 4. Reasons for any differences between observed and anticipated outcomes, including the influence of context 5. Costs and strategic trade-offs, including opportunity costs | Paragraphs 1-7 under the discussion |
| **16. Limitations** | 1. Limits to the generalizability of the work 2. Factors that might have limited internal validity such as confounding, bias, or imprecision in the design, methods, measurement, or analysis c. Efforts made to minimize and adjust for limitations | 1. Generatability is not directly relevant in this work as this was a pilot study. However, comparison with available studies is made in paragraph 4 of the discussion 2. Last paragraph of the discussion |
| **17. Conclusions** | 1. Usefulness of the work 2. Sustainability 3. Potential for spread to other contexts 4. Implications for practice and for further study in the field 5. Suggested next steps | Paragraphs 6,7 of the discussion  Conclusions section |
