# Supplementary material for: The Clean pilot study: evaluation of an environmental hygiene intervention bundle in three Tanzanian hospitals
Source: Antimicrob Resist Infect Control. 2021 Jan 7;10:8. doi: 10.1186/s13756-020-00866-8 (PMC7789081; doi:10.1186/s13756-020-00866-8)
Supplement: Supplementary file 3 — Additional file 3 “Key hand-touch sites”. Selection of hand-touch sites. [file 13756_2020_866_MOESM3_ESM.docx]

# Additional File III - Selection of high touch sites

Hand-touch sites are surfaces in the wards that are frequently touched, in particular by healthcare workers and thus pose a risk of pathogen cross-transmission. Studies in the UK and the US summarised the most frequently touched hand-touch sites. The hand touch sites near the patient pose the greatest risk of infection.

For this study we chose to focus on patients’ beds (mattress and frame – Note: we anticipate mattresses to be sampled less frequently because sampling is only possible when no patients or bedsheets are present on the bed). This was found to be an important hand touch site in the literature. In addition, we conducted ten 30 minutes observations to assess hand touch sites across the wards of interest in our study sites. For this observation, different wards were purposively sampled to provide a range of hospital environments. These observations took place during morning and afternoon shifts. Day and time of observation was chosen based on accessibility to the ward and logistic considerations. Beds were consistently the most touched area across the wards.

Bed frames and mattresses were also chosen based on the criteria listed in Table 1 which we used to select which main hand touch sites to sample.

Beyond bed frame and mattresses, we sampled four extra items in one facility as there was no sufficient number of beds: an equipment trolley, a bedside locker, a sink, and a water tap used for hand hygiene. These were also found to be key hand touch sites in our formative research.

Table 1 – selection criteria for choosing evaluation site (red = NO; grey = YES)

|  | Bed mattress | Bed frame | Trolley | Nurs.  Table |
| --- | --- | --- | --- | --- |
| 1. Minimal disruption of patient experience |  |  |  |  |
| 1. Key hand touch site across wards and thus poses risk to pathogen transmission to patients (within patient zone) |  |  |  |  |
| 1. Cleaned at least daily |  |  |  |  |
| 1. Sufficient in number in each ward |  |  |  |  |
| 1. Present consistently across wards to ease data collection |  |  |  |  |
| 1. Unlikely to move from room within the 6 months |  |  |  |  |
| 1. Needs to address cleaning behaviour |  |  |  |  |
| 1. Material OK for the use of gel dots | Cloth and patient movement makes it hard |  |  |  |
