# Supplementary material for: The Clean pilot study: evaluation of an environmental hygiene intervention bundle in three Tanzanian hospitals
Source: Antimicrob Resist Infect Control. 2021 Jan 7;10:8. doi: 10.1186/s13756-020-00866-8 (PMC7789081; doi:10.1186/s13756-020-00866-8)
Supplement: Supplementary file 4 — Additional file 4 “Sample size”. Sample size calculations. [file 13756_2020_866_MOESM4_ESM.docx]

# Additional File IV – Façade and Layout

1. **Table façade**


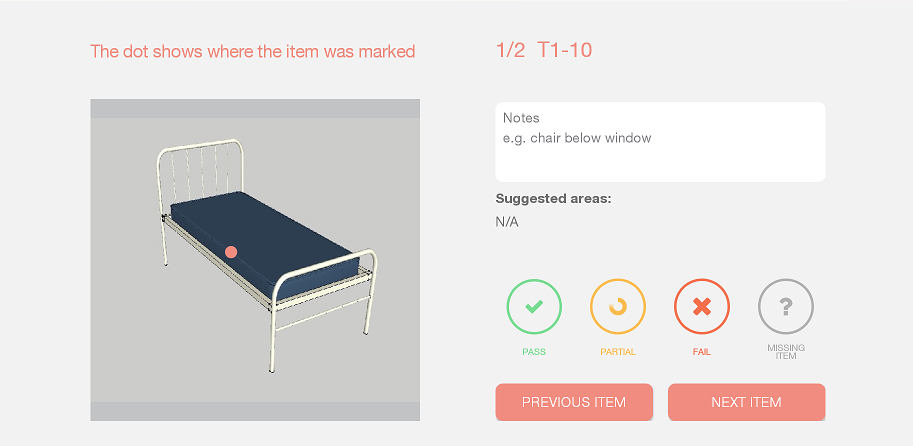


**B) Layout**

# Additional File VI – sample layout for data collection


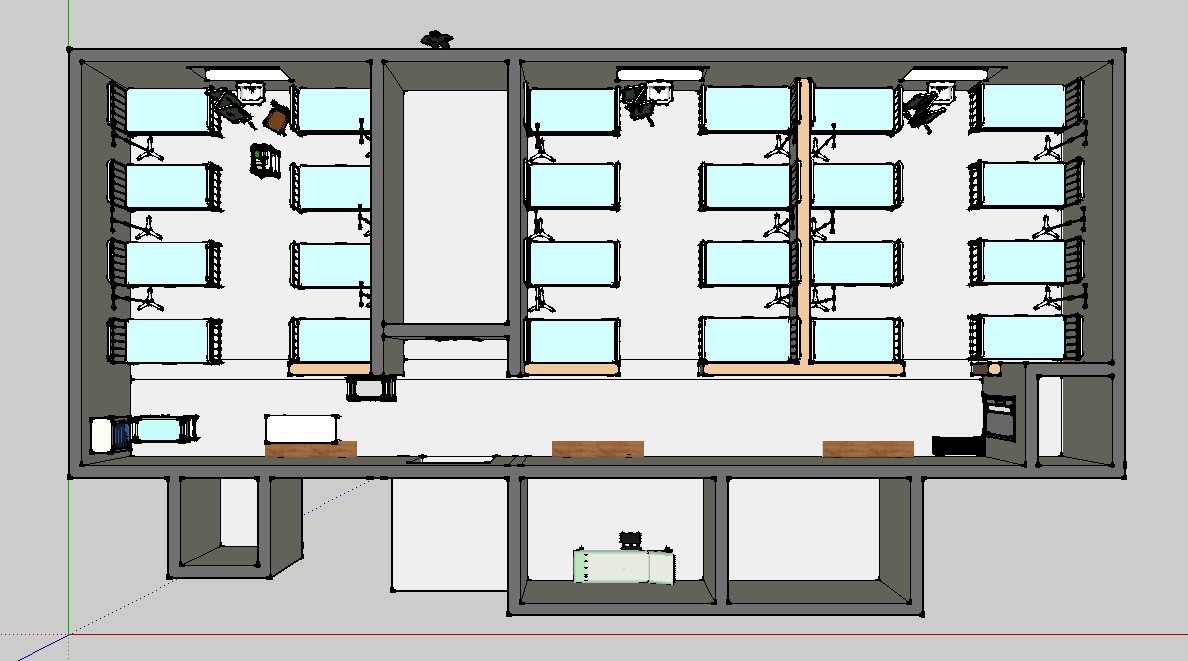


**1**

**2**

**3**

**4**

**6**

**5**

**7**

**8**

**9**

**10**
