# Supplementary material for: The Clean pilot study: evaluation of an environmental hygiene intervention bundle in three Tanzanian hospitals
Source: Antimicrob Resist Infect Control. 2021 Jan 7;10:8. doi: 10.1186/s13756-020-00866-8 (PMC7789081; doi:10.1186/s13756-020-00866-8)
Supplement: Supplementary file 5 — Additional file 5 “Tablet façade”. Visual of data collection tool. [file 13756_2020_866_MOESM5_ESM.doc]

# Additional File III – Sample topic guide for qualitative interviews

**Interview or FGD topic guide (champions/cleaners)**

**Expected duration: 45-60 minutes**

**Aims**

The aim of this interview is to investigate how trainers and healthcare workers (managers, nurses, ward attendants – anyone involved in environmental hygiene) feel and think about environmental hygiene and what contextual elements help or hinder this activity. **Note that environmental hygiene refers to both cleaning and disinfection, as well as waste disposal.**

Participants

| ID# | Gender | Age group | Job Title | Years at facility |
| --- | --- | --- | --- | --- |
|  |  |  |  |  |

Researcher name __________________________

Health facility number ________________________

Date |__|__/__|__/__|__|

Duration of interview

Location of interview

**Introduction**

I am ______________________________ from ______________________

- General purpose of the study (see additional text on separate page for below points)
- Aims of the interview and expected duration
- Who is involved in the process (other participants)
- Why the participant’s cooperation is important
- What will happen with the collected information and how the participant/target group will benefit
- Any questions?
- Consent

**Ensure all major topics are covered, but cover them in whatever order is natural. Feel free to follow up on anything interesting or unexpected.**

**Ensuring environmental hygiene in the facility**

What do you think helps your ability to conduct/ensure (use the word ensure: for managers/trainers) environmental hygiene? Anything else?

What barriers do you face in conducting/ensuring environmental hygiene? (Prompts: time, other patient caring activities, supplies, infrastructure, number of hand washing stations, water supply; quantity and quality, soap, waste disposal training/bins/equipment, workload, gender, staffing levels, knowledge, type of employment, conflict resolution, supervision, motivation etc.)

Considering the barriers you have just described, which three do you consider to be the biggest (of high priority) and hence needing urgent action?

Do you think your colleagues respect the role of cleaning?

Are other priorities competing with these environmental tasks? (What other activities do you carry out in the maternity ward apart from cleaning; routinely and/or when necessary? Prompts: injections, dressing, drug dispensing, delivery,). Which ones and how often?

You received the training of trainers at MUHAS in January 2019,– what did you learn during the training. What do you remember most about the training? How different was it from what you did before?

Do you think anything has changed since the training in this facility? If so, what? And why?

Have you heard of the cleaning champions? What do you think about them (if they are champions – ask what they think about their role)? What makes them good champions? What should be the qualities of an ideal champion?

Were you able to implement what you learnt during training on the ward? What made that easier? What made that difficult?

If you are interviewing a trainer or champion please also ask: Did you provide training on environmental hygiene in the past few months? What did you think about the training (what was good? What was less good?)?

Has there been a fresher training in your facility? When? Who facilitated it? What was the content of the refresher training? What did you think about the training (what was good? What was less good?)?

Have you received any kind of supervision since the trainings? From who? What do you think about the supervision, (what was good? What was less good?)?

How are you planning to give ongoing supervision to people that you trained (if you are interviewing champions)

What was your experience about people being able to change their practice after the training? Did people discuss problems they would face, did you come across problems. What do you think makes people change their practice? What do you think motivates people.

If this training would need to be delivered elsewhere, what would you change to ensure the success of the training? What would you do to help people implement what they learnt in training?

**Thank the respondents for their time. Enter the time interview ended in the relevant boxes at the beginning of the questionnaire**

# Topic guide for an in-depth interview with training college

**Expected duration: 45-60 minutes**

**Aims**

The aim of this interview is to investigate how trainers and healthcare workers (managers, nurses, ward attendants – anyone involved in environmental hygiene) feel and think about environmental hygiene and what contextual elements help or hinder this activity. **Note that environmental hygiene refers to both cleaning and disinfection, as well as waste disposal.**

Participants ID#

Researcher name __________________________

Date |__|__/__|__/__|__|

Duration of interview

Location of interview

**Introduction**

I am ______________________________ from ______________________

- General purpose of the study (see additional text on separate page for below points)
- Aims of the interview and expected duration
- Who is involved in the process (other participants)
- Why the participant’s cooperation is important
- What will happen with the collected information and how the participant/target group will benefit
- Any questions?
- Consent

We are hoping to get funds to repeat this project on a larger scale. To help us do this could you tell us three things that you liked about the project – and three things that you think we should change in the future?

If these things are not covered in what they liked and what they didn’t like – probe on the following

- What was their experience in adapting the tool?
- What was their experience in doing/conducting the training?
- What was the experience supporting the training in the facilities?

What were the differences and similarities between the training in the three different hospitals and why do you think this happened?

The training manual included a module on supportive supervision – for the facility champions to follow up with the cleaners at their facility. What did they think of that module? Do they think that supervision of cleaners needs to be improved?

**Thank the respondents for their time. Enter the time interview ended in the relevant boxes at the beginning of the questionnaire**
