# Supplementary material for: The Clean pilot study: evaluation of an environmental hygiene intervention bundle in three Tanzanian hospitals
Source: Antimicrob Resist Infect Control. 2021 Jan 7;10:8. doi: 10.1186/s13756-020-00866-8 (PMC7789081; doi:10.1186/s13756-020-00866-8)
Supplement: Supplementary file 6 — Additional file 6 “Layout”. Example of ward layout for data collection. [file 13756_2020_866_MOESM6_ESM.docx]

# Additional File VI – Sample size

Details of sample size calculations

- Each surface is considered a “cluster”
- 10 surfaces per ward – for a total of 40 surfaces per hospital
- We aimed to collect in total 20 samples per surface; 10 before the intervention across 14 weeks and 10 after the intervention across 14 weeks
- This translates into 400 observations to be collected before and 400 after the intervention in each hospital.
- Our baseline predictions for cleaning behaviour are low. In the formative phase in two regions of Tanzania the proportion of delivery beds was 15% (6/7 delivery beds were microbiological clean). In Table 2 we have different scenarios with baseline % varying from 20% to 40%
- We used the sample size formula for cluster-randomized trials to estimate which power scenarios we could expect from our sample size. We had at least 70% power to detect the scenarios shaded in grey in table A below.

Table A – Sample size scenarios

| Number of surfaces per cluster | Clusters per facility | Baseline proportion | Post-int. proportion | ICC | Power |
| --- | --- | --- | --- | --- | --- |
| 10 | 40 | 20% | 30% | 0.01 | 97% |
| 10 | 40 | 20% | 30% | 0.15 | 73% |
| 10 | 40 | 40% | 50% | 0.01 | 77% |
| 10 | 40 | 40% | 50% | 0.15 | 45% |
