# Supplementary material for: The Clean pilot study: evaluation of an environmental hygiene intervention bundle in three Tanzanian hospitals
Source: Antimicrob Resist Infect Control. 2021 Jan 7;10:8. doi: 10.1186/s13756-020-00866-8 (PMC7789081; doi:10.1186/s13756-020-00866-8)
Supplement: Supplementary file 7 — Additional file 7 “Sensitivity analyses”. Sensitivity analyses results for microbiological cleanliness. [file 13756_2020_866_MOESM7_ESM.docx]

# Additional File VII - Sensitivity analyses for weekly change in odds of cleanliness (ACC)

We conducted two sensitivity analyses for the main impact outcome (ACC pass/fail) using the same model which gave results to Table 3 in the manuscript : a) re-calculating bed occupancy at one facility excluding infrequently used delivery beds, and b) restricting analyses to data collected from bedframes in case staff might engage with cleaning frames and mattresses differently.

| **Microbiological cleanliness (ACC<2.5cfu/cm^2^)** | **n** | **Crude odds ratio (CI)** | **p-value** | **N** | **Adjusted odds ratio (CI)** | **p-value** |
| --- | --- | --- | --- | --- | --- | --- |
| *Analyses with bed occupancy at one facility excluding infrequently used delivery beds* |  |  |  |  |  |  |
| Pre-training | 361 | 1.22 (1.04-1.44) | 0.014 | 355 | 1.32 (1.10-1.58) | 0.003 |
| Post-training | 667 | 1.08 (1.04-1.12) | <0.001 | 651 | 1.08 (1.04-1.13) | <0.001 |
| *Analyses restricted to frame only* |  |  |  |  |  |  |
| Pre-training | 264 | 1.15 (0.97-1.35) | 0.098 | 258 | 1.27 (1.05-1.55) | 0.014 |
| Post-training | 451 | 1.10 (1.05-1.15) | <0.001 | 435 | 1.08 (1.04-1.13) | <0.001 |
