# Supplementary material for: The Clean pilot study: evaluation of an environmental hygiene intervention bundle in three Tanzanian hospitals
Source: Antimicrob Resist Infect Control. 2021 Jan 7;10:8. doi: 10.1186/s13756-020-00866-8 (PMC7789081; doi:10.1186/s13756-020-00866-8)
Supplement: Supplementary file 8 — Additional file 8 “ACC breakdown”. Results by categories of ACC. [file 13756_2020_866_MOESM8_ESM.docx]

# Additional File VIII– Categorical classification of cleanliness (ACC) by study period

| **CFU enumerated**  **from dipslide** | **Pre-training, n (%)**  **N = 366** | **Post-training, n (%)**  **N = 672** |
| --- | --- | --- |
| No growth | 11 (3.0) | 119 (17.7) |
| >0 to <2.5 | 58 (15.9) | 153 (22.8) |
| 2.5 to <12 | 121 (33.1) | 283 (42.1) |
| 12 to <40 | 113 (30.9) | 99 (14.7) |
| 40+ | 58 (15.9) | 14 (2.1) |
| Missing | 5 (1.4) | 4 (0.6) |
