# Supplementary material for: The Clean pilot study: evaluation of an environmental hygiene intervention bundle in three Tanzanian hospitals
Source: Antimicrob Resist Infect Control. 2021 Jan 7;10:8. doi: 10.1186/s13756-020-00866-8 (PMC7789081; doi:10.1186/s13756-020-00866-8)
Supplement: Supplementary file 9 — Additional file 9 “Sensitivity and Specificity”. Sensitivity and specificity calculations. [file 13756_2020_866_MOESM9_ESM.docx]

# Additional File IX – sensitivity and specificity details

The False Negative (FN) rate is particularly high (51%). This was true across intervention period, hospital, ward type, audit location, and shift type, with the FN rate ranging from 47% (bedframe audit location; postnatal vaginal ward type) to 63% (mattress audit location). Interestingly, the FN rate went from 55% in the pre-intervention period to 48% in the post-intervention period, while the TN rate when from 12% to 33%.

The associated sensitivity, specificity, positive predictive values (PPV), and negative predictive values (NPV) are shown in Table 2 across various study characteristics. Overall, the gel dot sensitivity was very low, with 22% overall sensitivity (ranging from 6% to 32% across study characteristics) (Table 2). The specificity was moderate (overall 80%; ranging from 62% to 91%).

For the interpretation, it is important to remember that the gel dots and the dipslides results measure *cleaning* during two slightly different time frames. Time of application of gels dots was randomly allocated to either morning, afternoon or night shift. Gel dot removal was measured the next day, after general cleaning was supposed to take place (between 8AM and 10AM). Whereas dipslides were only measured after general cleaning was supposed to take place (between 8AM and 10AM) concurrently to gel dots measurement. Therefore, gel dots reflect any cleaning actions between application and the time of measurement; whereas, dipslides reflect cleaning around the time of measurement only. Indeed, as our results show in the manuscript microbiological cleanliness, measured with dipslides tends to be lower than cleaning actions measured with gel dots.

*Table 1: True positive/negative and false positive/negative rates for paired gel dots/dipslides.*

| **Overall**  n = 1185 | | **Dipslides** | |  |
| --- | --- | --- | --- | --- |
|  |  | **+** | **─** | **% total** |
| **Gel dots** | **+** | 15%  TP^1^ | 7%  FP^1^ | 22% |
|  | **─** | 51%  FN^1^ | 27%  TN^1^ | 78% |
|  | **% total** | 66% | 34% | 100% |
| ^1^ TP = true +; TN = true ─ ; FP = false +; FN = false ─ | | | | |

*Table 2: Sensitivity, specificity, positive predictive value, negative predictive value of gel dots across various study characteristics*

|  | Sensitivity | Specificity | PPV^1^ | NPV^1^ |
| --- | --- | --- | --- | --- |
| Overall | 22% | 80% | 68% | 34% |
| Hospital |  |  |  |  |
| 1 | 18% | 87% | 76% | 31% |
| 2 | 26% | 73% | 64% | 35% |
| 3 | 23% | 78% | 65% | 38% |
| Intervention period |  |  |  |  |
| Pre-intervention | 32% | 62% | 78% | 18% |
| In-between | 6% | 91% | 50% | 39% |
| Post-intervention | 19% | 82% | 60% | 41% |
| Audit location type |  |  |  |  |
| Bedframe | 25% | 78% | 65% | 39% |
| Mattress | 14% | 87% | 75% | 27% |
| Ward type |  |  |  |  |
| Postnatal c-section | 20% | 82% | 64% | 39% |
| Postnatal vaginal | 25% | 86% | 75% | 41% |
| Labour | 15% | 75% | 57% | 29% |
| Neonatal | 29% | 72% | 73% | 28% |
| Shift |  |  |  |  |
| Morning | 16% | 84% | 66% | 33% |
| Afternoon | 27% | 76% | 69% | 34% |
| Night | 25% | 79% | 69% | 38% |
|  |  |  |  |  |
| ^1^ PPV = positive predictive value; NPV = negative predictive value | | | | |
