# Supplementary material for: The Clean pilot study: evaluation of an environmental hygiene intervention bundle in three Tanzanian hospitals
Source: Antimicrob Resist Infect Control. 2021 Jan 7;10:8. doi: 10.1186/s13756-020-00866-8 (PMC7789081; doi:10.1186/s13756-020-00866-8)
Supplement: Supplementary file 10 — Additional file 10 “Champions breakdown”. Details of champions selected. [file 13756_2020_866_MOESM10_ESM.docx]

# Additional File X – Champions breakdown

| **Cadre (In charge of)** | **Hospital 1** | **Hospital 2** | **Hospital 3** |
| --- | --- | --- | --- |
| Registered nurse (Labour ward) | 1 |  |  |
| Assistant Nurse Officer (Labour ward) |  | 1 | 1 |
| Nurse Officer (Antenatal ward) | 1 |  |  |
| Environmental Health Officer | 1 | 1 | 1 |
| Nurse Officer (post-natal C-section ward) | 1 |  |  |
| Nurse Officer (post-natal vaginal ward) | 1 |  |  |
| Assistant Nurse Officer (post-natal C-section ward) |  | 1 | 1 |
| Assistant Nurse Officer (post-natal vaginal ward) |  | 1 |  |
| Enrolled nurse (Kangaroo ward) |  | 1 |  |
| Nurse officer (Maternity block) |  | 1 | 1 |
| Nurse officer (Neonatal Ward) | 1 |  | 1 |
| **Total** | **6** | **6** | **6** |
